# Repeatability, reproducibility, and comparison of ocular biometry using a new optical coherence tomography-based system and another device

**DOI:** 10.1038/s41598-020-71192-7

**Published:** 2020-09-02

**Authors:** Adam Wylęgała, Bartłomiej Bolek, Robert Mazur, Edward Wylęgała

**Affiliations:** 1 Ophthalmology Department, Railway Hospital, Panewnicka 65, 40-765 Katowice, Poland; 2 II School of Medicine with the Division of Dentistry, Zabrze Medical University of Silesia, Katowice, Poland

**Keywords:** Medical research, Translational research

## Abstract

Precise measurement of axial length before cataract surgery is necessary for the proper lens implantation. We included 100 eyes of 56 patients in the study. The mean age was 41 (15–84 years). Measurements of axial length, anterior chamber depth (ACD) carried out with the new Revo NX were compared with those obtained with the IOLMaster 500. Interoperator testing was performed on 56 eyes of 56 participants. To test repeatability, axial length, ACD, central corneal thickness, and lens thickness were used. Inter-operator reproducibility was also assessed. The results were evaluated using Bland–Altman analyses. The mean ACD measured with the IOLMaster and Revo NX was 3.41 and 3.40 mm *p* = 0.467, respectively. The interclass correlation was excellent at the level of 0.975. ICC for axial length values was 0.999, and the mean was equal to 23.47 for the new device and 23.48 for IOLMaster. ICC for Inter-operator repeatability was higher than 0.99 in all parameters. Revo NX showed a very high level of repeatability with ICC ranging from 0.9929 for central corneal thickness to 0.9997 for axial length. Both devices showed excellent agreement and differences that are insignificant, which suggests that they can be used interchangeably.

## Introduction

Surgery is the only currently known way of treating cataracts. During the procedure, the opacified lens is extracted, and an intraocular lens is inserted. The power of the intraocular lens must be calculated before the surgery using the mathematical formulas that usually require axial length (AL), corneal power, anterior chamber depth (ACD). Failed measurement can lead to incorrect implantation resulting in postoperative refractive error^1^. Currently, the AL and ACD can be measured with ultrasounds, partial coherence interferometry, optical low-coherence reflectometry, and optical coherence tomography. The primary advantage of optical coherence tomography-based systems is that they allow visualization of the entire eye. Hence any abnormalities which might affect calculations such as posterior staphyloma, lens tilt, macular edema, or poor fixation can also be diagnosed^2^. Spectral-domain optical coherence tomography (SD-OCT) is a powerful optical coherence tomography technology that has been in use to image both the anterior and posterior segments^3–5^. Recently with the introduction of IOLMaster 700 (Carl Zeiss Meditec, Jena, Germany) the importance of visualization of the entire AL during intraocular lens calculations has been shown^2^. However, IOLMaster 700 provides only screening quality and can only obtain the image of a small area (1 mm width) around the macula. The huge advantage of an optical coherence tomography-based system is that it can be used as both the imaging device that can produce cross-sections of the anterior segment and the retina, oct angiography and measure values such as macular or retinal nerve fiber layer thickness while still functioning as a biometer used to calculate the lens implantation. Another application of AL measurement is to monitor patients with high myopia. While ACD evaluation can act as a screening tool for glaucoma^6^. Revo NX (Optopol Technology Ltd, Zawiercie, Poland) is the first OCT system, that can measure AL, ACD, lens thickness, and with the use of add on lens it can visualize the anterior chamber and measure anterior and posterior curvature of the cornea.

The introduction of a new measuring device requires a study that will evaluate its accuracy and checking whether the values provided by the new device correlate with the gold standard. In 1986 Bland and Altman established a graphical technique that facilitates the comparison of new and old methods^7^. There are two forms of measuring the precision of a device: repeatability and reproducibility. Repeatability means variability of results measured in short intervals, while reproducibility is defined as variability of results measured under different circumstances, e.g., exams taken by different operators^7^. Several studies have already compared the IOLMaster 500 and other devices^1,2,8^. It is the first known study to show the comparison with the Revo NX and IOLMaster 500.

## Methods

This prospective study followed the tenants of the Helsinki Declaration and was approved by the bioethical committee of Silesian Medical University. All participants signed the informed consent and obtained a leaflet explaining the nature of the study. Informed consent was taken from the guardians of the minors included in the study. Before the measurements, patients had a thorough ophthalmic examination including best-corrected visual acuity, non-contact tonometry with Corivs ST (Oculus, Weltzar, Germany) slit lamp and fundus examinations. Participants with no signs of ocular disease that can lead to unstable AL values such as retinal detachment, intravitreal hemorrhage were excluded from the study.

### Instruments

IOLMaster 500 (Carl Zeiss Meditec, Oberkochen, Germany) utilizes partial light interferometry to obtain AL while keratometry is measured using six light points on the cornea within 2.3 mm diameter. This machine emits 780 nm infrared light, ACD is measured using the Scheimpflug principle with the use of lateral slit illumination of 590 nm wavelength^9,10^.

Revo NX software version 9.0.0 (Optopol Technology, Zawiercie, Poland) is a new posterior segment optical coherence tomography instrument with an add on for measuring the anterior segment as well. The device is equipped with a biometry module. It operates at 830 nm center wavelength with 18 µm transverse and 5 µm axial imaging resolutions, scanning speed 110,000 A-scan/s. Furthermore, the device can also measure total curvature of the cornea, ACD, AL, lens thickness, and central corneal thickness. Revo NX can create topography maps and calculated anterior and posterior K1/K2 with an add on lens and Topo software (Optopol technology). Older devices used light interferometry to determine AL; however the AL can be calculated using a B scan as well. The device will scan the eye in four different locations along the visual axis. First, the scanning beam will determine the anterior and posterior surface of the cornea. Then both surfaces of the lens will be measured, and finally, the posterior surface of the retina will be delineated. B-scanning along the z-axis requires a shift of the c-gate and the internal optics positions, which are done automatically. This instrument takes a series of 10 measurements in the x and y-axis, which is followed by the calculation of the mean value. All 10 measurements are displayed on the screen as an optical coherence tomography cross-section through the eye and can be verified by the technician.

Both devices were installed in the same darkened and air-conditioned room. Each patient had their measurements taken on the same morning between 8:00 a.m. and 12:00 a.m.

### Repeatability and inter-operator reproducibility

To test if the measurements were stable, 60 eyes of 60 participants were examined using Revo NX. After quality check 56 eyes of 56 participants were analyzed. To avoid bias, only one eye per patient was enrolled. Six consecutive measurements were retaken to test the repeatability of each patient. Two operators, A (Bartłomiej Bolek) and B (Robert Mazur), made six measurements each (12 in total). Later these values were compared to test inter-operator reproducibility.

### Comparison between Revo NX and IOLMaster 500

After an eligibility check, 56 of 65 Patients (100 of 113 eyes) were enrolled in the final analysis. In the case of four patients (five eyes) with severe cataract measurements with neither devices were possible. Nevertheless, for five other patients (eight eyes) with severe cataract measurement was possible on the REVO NX only. Fifty-two right eyes and 48 left eyes were included. The mean age of the subjects was 41 ± 19.77 years (15–84 years). The majority of eyes (85) showed no signs of ocular diseases. Seven of them had cataracts, one had geographic atrophy, five had acrylic, and one silicone intraocular lens, and one eye had a history of penetrating keratoplasty.

### Statistical analysis

Statistical analysis was conducted—using Statistica software ver. 13.1 (Dell Inc, Tulsa, OK, USA.). A comparison between the new device and the IOLMaster 500 was analyzed using Bland–Altman plots. The normality of the data was checked using the Shapiro–Wilk test. The paired Student t-test was used to evaluate differences in ACD between biometers. While to assess significance in AL measured by tested devices—Wilcoxon Matched Pairs test was applied. .

## Results

### Repeatability and reproducibility

The central corneal thickness, AL, ACD, and lens thickness were compared between two operators under the same circumstances. Mean corneal thickness measured by operator A was 0.5481 ± 0.038 mm and the value measured by the other observer was 0.54795 ± 0.038 mm. The mean difference was − 0.0001 mm. (Table 1). The mean lens thickness measured by operator A was 4.0681 mm, while the value measured by the other operator was 4.0666 mm. Mean AL was 23.5223 mm measured by operator A and 23.5248 mm by operator B. ACD was also evaluated. Operator A and B achieved similar results—3.4376 mm and 3.4402 mm, respectively.

**Table 1:** Coefficient of inter-operator reproducibility and repeatability of central corneal thickness and anterior chamber depth, axial length and lens thickness measurements for different operators.

	Operator	Mean (mm)	Std.dev	Sw	TRT	CoV (%)	ICC
**Central corneal thickness**							
Repeatability	A	0.5464	0.0407	0.0035	0.0096	0.6325	0.9929
Repeatability	B	0.5463	0.0413	0.0030	0.0082	0.5413	0.9950
Reproducibility		0.5464	0.0409	0.0032	0.0088	0.5783	0.9942
**Lens thickness**							
Repeatability	A	4.0708	0.4000	0.0366	0.1014	0.8990	0.9918
Repeatability	B	4.0737	0.3953	0.0334	0.0925	0.8199	0.9930
Reproducibility		4.0723	0.3966	0.0357	0.0989	0.8765	0.9921
**Axial length (AL)**							
Repeatability	A	23.5328	1.0414	0.0167	0.0463	0.0711	0.9997
Repeatability	B	23.5333	1.0427	0.0171	0.0473	0.0726	0.9997
Reproducibility		23.5326	1.0397	0.0169	0.0468	0.0718	0.9997
**Anterior chamber depth (ACD)**							
Repeatability	A	3.4097	0.3985	0.0202	0.0558	0.5913	0.9975
Repeatability	B	3.4112	0.3966	0.0273	0.0757	0.8008	0.9953
Reproducibility		3.4105	0.3967	0.0236	0.0653	0.6911	0.9966
Number of eyes	56						
Number of participants	56						

*Std.dev* standard deviation, *Sw* within-subject standard deviation, *TRT* test–retest repeatability, *CoV* within-subject coefficient of variation, *ICC* intraclass correlation coefficient.

### Inter-operator reproducibility

All tested parameters had excellent ICC. The highest level of repeatability and reproducibility was observed in AL measurements. Observer A repeated of 0.9997, and B 0.9997. The total reproducibility was 0.9997. The lowest ICC value was obtained in central corneal thickness measurements performed by operator A, and A was 0.9929 and B 0.9950, respectively, with reproducibility of 0.9942.

### Comparison

**Figure 1:**
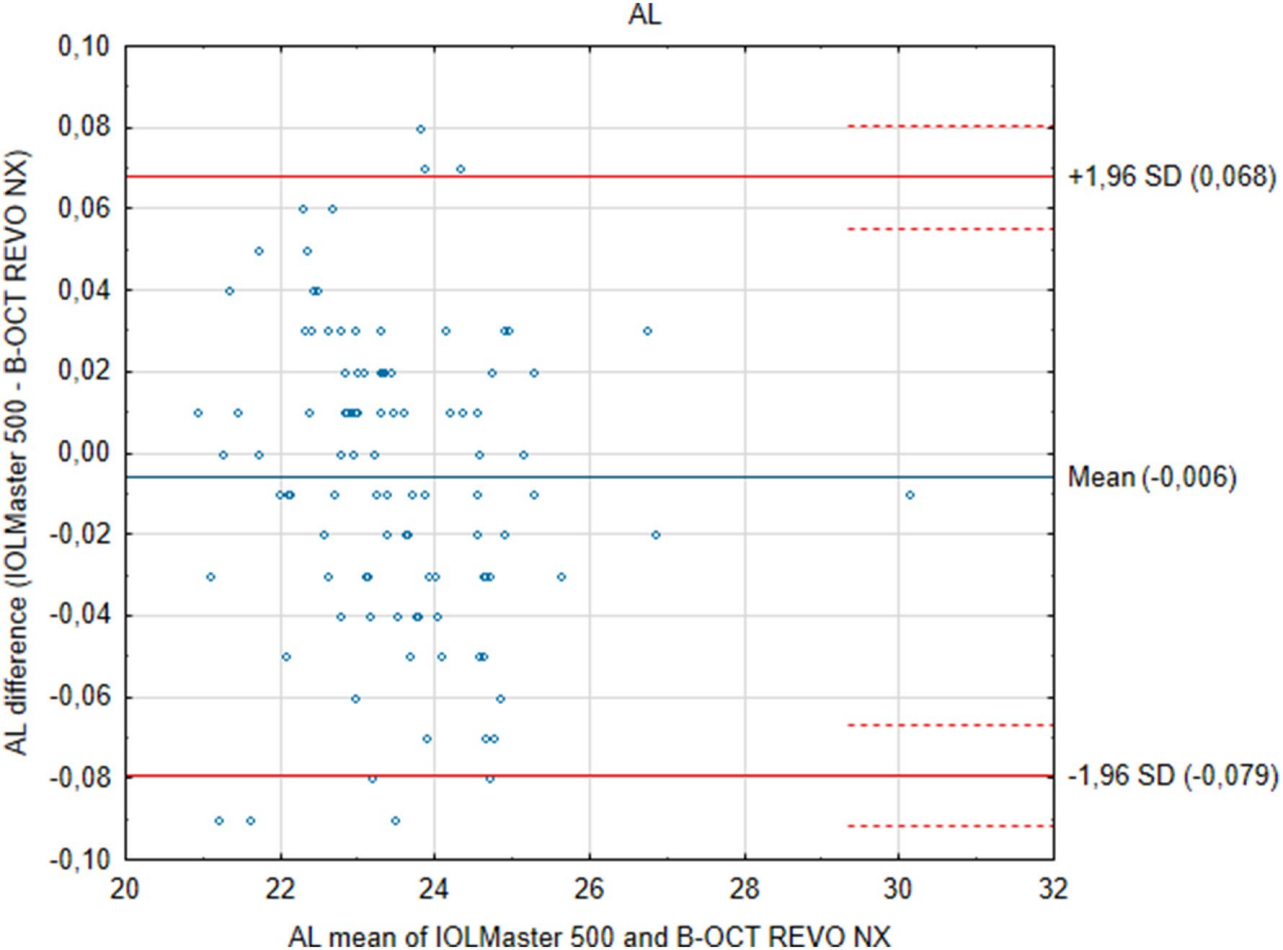
Bland–Altman plots show the agreement between the Revo NX Spectral Domain optical low coherence tomographer and the IOLMaster 500 partial coherence interferometry for measurements of the axial length (AL). The mean difference is represented by the solid blue line whereas the dotted lines represent ± 1.96 SD.

**Figure 2:**
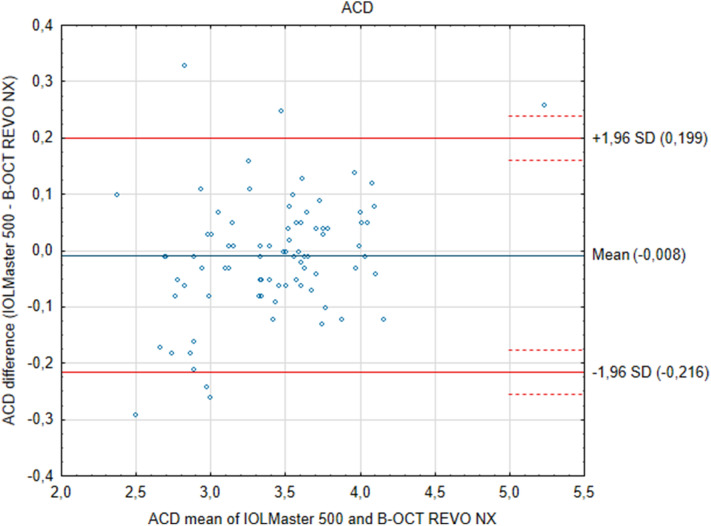
Bland–Altman plots show the agreement between the Revo NX spectral domain optical low coherence tomographer and the IOLMaster 500 partial coherence interferometry for measurements of the anterior chamber depth (ACD). The mean difference is represented by the solid blue line whereas the dotted lines represent ± 1.96 SD.

The results of measurements from the two devices show that the differences between them are insignificant. ACD measurements performed with the Revo NX and the IOLMaster showed a narrow 95% limits of agreement, which implies good agreement. Similarly, the value range and 95% limits of agreement were more extensive for axial length values between the two tested systems. Further, the Bland–Altman plots showed no specific trends. ACD measured in pseudophakic eyes was 0.13 mm smaller when measured with IOLMaster *p* = 0.408, while in phakic eyes, it was only 0.0049 mm smaller *p* = 0.72. The mean AL, as measured with the new device, was 23.475 ± 1.34 mm, while with the IOLMaster 500 it was 23.481 ± 1.34 mm and did not show statistical difference* p* = 0.140 (Fig. 1). ICC for AL was 0.999. Mean ACD as measured by the new device and IOLMaster was 3.407 ± 0.486 mm and 3.416 ± 0.450 mm respectively, and again the differences were insignificant *p* = 0.4665, ICC was 0.975 (Fig. 2). The mean difference between Revo NX and IOLMaster 500 in AL was − 0.0058 mm, and in ACD it was − 0.0085 mm. Both devices showed a narrow 95% CI (Table 2).

**Table 2:** Differences in axial length and anterior chamber depth between the devices.

	Estimate	Lower endpoint of 95% CI	Upper endpoint of 95% CI
**Anterior chamber depth (ACD) 100 eyes**			
Mean difference mm	− 0.0085		
Standard deviation	0.1059		
Lower limit of agreement	− 0.2160	− 0.2549	− 0.1771
Upper limit of agreement	0.1991	0.1602	0.2380
**ACD IOL 6 eyes**			
Mean difference	0.1300		
Standard deviation	0.2166		
Lower limit of agreement	− 0.2945	− 0.7773	0.1883
Upper limit of agreement	0.5545	0.0717	1.0373
**ACD Phakic 94 eyes**			
Mean difference	0.0049		
Standard deviation	0.0883		
Lower limit of agreement	− 0.1682	− 0.2137	− 0.1228
Upper limit of agreement	0.1780	0.1325	0.2235
**Axial length (AL) 100 eyes**			
Mean difference mm	− 0.0058		
Standard deviation	0.0375		
Lower limit of agreement	− 0.0794	− 0.0920	− 0.0667
Upper limit of agreement	0.0678	0.0551	0.0804

*CI* confidence interval.

## Discussion

The recent clinical introduction of OCT-based biometers enabled identifying any irregularities in the eye anatomy such as lens tilt, macular edema, or central retinal detachment^11^. In this article, we used this new technology and analyzed the parameters in several significant ways. Firstly, using Revo NX, we showed a high level of intraoperator repeatability. Secondly, we tested measurements conducted by two operators. Finally, Revo NX and IOLMaster 500 were compared and showed good agreement.

Previous studies showed that the IOLMaster 500 has a high rate of correlation with the newer model of 700 with insignificant differences. One of the difference is the ability to penetrate denser cataracts, making it possible for a Swept-source light to measure through media opacities^2,11,12^. IOLMaster 500 was shown to be precise in both intersession and intrasession tests^13^. A high interclass correlation of 1 when measuring AL between IOLMaster 500 and 700 was observed. The mean difference between ACD measured by the two devices was only 0.08 mm, which is not clinically significant^2,11^. Another study assessed differences between IOLMaster 500 and Scheimpflug based camera Galileli G6 (Zimer, Port, Switzerland). The two devices did not vary concerning AL, K, and ACD. However, the authors concluded that they should not be used interchangeably^14^. Carkeet et al.^15^ compared IOLMaster 500 and ultrasound biometry in children. The authors noted that the repeatability of the light-based method is significantly higher than the ultrasound-based. They conclude that partial coherence tomography is a superior method for AL testing due to its high accuracy, noninvasiveness, and ease of application. Similar results were published by Lam et al.^16^ IOLMaster 500 was also compared with the Argos Swept-Source optical biometer. Although the differences in AL were significant, the results varied from 0.05 to 0.01 mm, making them clinically insignificant^17^.

It has to be noted that AL measurement is the most critical step in assessing intraocular lens power, and it accounts for approximately 50% of predicted error in IOL power calculation^13^. The mean difference of − 0.006 mm in AL presented in this study would correspond to 0.016–0.026 D in IOL power calculation following the SRK-T formula, depending on the AL of the eye^18^. Such a difference is probably not clinically relevant and will not affect postsurgical visual outcomes. Secondly, AL measurement can be used to monitor the progression of myopia. In the past three decades, the prevalence of Myopia has approximately doubled^19^. Currently, two methods for measuring AL and ACD are used. Partial coherence interferometry or optical low coherence reflectometry. IOLMaster 500 uses partial coherence interferometry to obtain measurements^2^. We observed that more eyes with dense cataracts were successfully measured using Revo NX. The use of light source with longer wavelength compared with IOLMaster lead to a better penetration through dense cataracts. As IOLMaster 500 was shown to produce a reduced level of reproducibility of ACD in pseudophakic eyes^20^, we decided to present results separately of phakic and pseudophakic eyes.

Another group assessed the comparability of the IOLMaster 500 and Galilei G6. Those devices gave similar AL results and ACD results^14^. The agreement between devices is high and the differences are probably due to the diverse methods utilized.

The knowledge of measurement reliability can help physicians to determine if a specific device is of any value. ICC can be used as a factor contributing to show if the test has probative value^21^.Sikorski and Suchoń^22^ compared IOLMaster 700 with Revo NX and showed excellent ICC for AL = 1.00 for central corneal thickness = 0.933; for ACD = 0.933; and for lens thickness = 0.985. The intraobserver repeatability (ICC ranged from 1.000 to 0.993. The authors enrolled 349 eyes into the study^22^. Another study compared the use of Lenstar 900 and Revo NX. Kanclerz et al. compared AL measured by Revo NX with an optical low-coherence reflectometer. The authors presented lower ICC from 0.92–0.986 for all tested parameters compared to our study. We can hypothesize that the discrepancies between our study or Sikorski’s^22^ are the result of different software versions^23^.

As biometry is used to calculate the IOL, the measurements must not lead to the implantation of a wrong IOL. It is especially important with the increase in the use of more complex IOL, such as multifocal or aspheric.

### Limitations

A potential limitation to this study is first that K readings were not compared, and secondly excluded eyes with dense cataracts and other media opacities were excluded. Moreover, the youngest participant was 15 years old. As the new device may be used to monitor the progression of myopia, further analysis is needed with much younger participants. We did not compare IOL powers as tested software version is not equipped with inbuilt IOL formulas.

## Conclusions

The repeatability and reproducibility of the Revo NX are excellent. In clinical practice, the IOLMaster 500 and the new device can be used interchangeably for AL, and ACD measurements.

## Supplementary information

Supplementary Information 1.

## Data Availability

Statistical data in the form of HTML web page including raw data is available in the Mendeley Repository https://dx.doi.org/10.17632/kwv73shvxh.2
