# Supplementary material for: Repeatability, reproducibility, and comparison of ocular biometry using a new optical coherence tomography-based system and another device
Source: Sci Rep. 2020 Sep 2;10:14440. doi: 10.1038/s41598-020-71192-7 (PMC7467923; doi:10.1038/s41598-020-71192-7)
Supplement: Supplementary file 1 — Supplementary Information 1. [file 41598_2020_71192_MOESM1_ESM.docx]

**Repeatability, Reproducibility, and Comparison of Ocular Biometry using a new Optical Coherence Tomography-based System and another Device.**

Running head: Comparison of two optical biometers

Adam Wylęgała^a^, Bartłomiej Bolek^a^, Robert Mazur^a^, Edward Wylęgała^a^,

^a^ Ophthalmology Department, Railway Hospital, Katowice, Panewnicka 65. 40-765 Katowice, Poland. II School of Medicine with the Division of Dentistry in Zabrze Medical University of Silesia, Katowice,

Running head: Comparison of two optical biometers.

Correspondence to

Adam Wylegala M.D. Ph.D.

[Adam.wylegala@gmail.com](mailto:Adam.wylegala@gmail.com)

Opthalmology Departament,

Railway Hospital, Katowice

Panewnicka 65. 40-765 Katowice, II School of Medicine with the Division of Dentistry in Zabrze Medical University of Silesia, Katowice,

ORCID: [**0000-0001-7295-4936**](https://orcid.org/0000-0001-7295-4936)

Numerical results for repeatability resp. reproducibility contain six quantities computed for observers separately resp. for the entire dataset:

- Mean
- Standard deviation
- Sw
- TRT
- CoV[%]
- ICC

**Mean** is the arithmetic mean of input values.

**Standard deviation** is the sample standard deviation, ie. with N-1 in the denominator, where N is the sample size.

**Sw** = within-subject standard deviation, is the root mean square of sample standard deviations of values measured on a single object, ie.

Sw = ((σ_1_^2^+...+σ_M_^2^)/M)^1/2^,

where M is the number of objects (eyes) and σ_k_ equals the sample standard deviation of values measured on the k-th object.

**TRT** = test-retest repeatability, is defined as = 2,77·Sw.

**CoV** = within-subject coefficient of variation, is defined as = Sw/Mean or = 100·Sw/Mean when reported as %.

**ICC** = intraclass correlation coefficient, is defined as the ratio of appropriate estimated variances. For this, it is assumed that there is a set of measured values y_ij_ for the i-th object in j-th repetition; i = 1, 2, ..., N (N = the number of objects) and j = 1, 2, ..., N_i_ (different numbers of repetitions are permitted for different objects). The measured values are modelled by the equation below:

y_ij_ = μ + σ_A_e_i_ + σ_B_e_ij_,

where μ is the average value and e's are independent realizations of a standard normal random variable and σ_A_^2^ and σ_B_^2^ are resp. interclass and intraclass variances. ICC is given by:

ICC = s_A_^2^/(s_A_^2^ + s_B_^2^),

where s_A_^2^, s_B_^2^ are estimated values of the variances σ_A_^2^, σ_B_^2^ according to the equations:

Σ_i_Σ_j_(y_ij_ - y_i_)^2^ = (M - N)s_B_^2^,

Σ_i_(y_i_ - y)^2^ = (N - 1)(s_A_^2^ + s_B_^2^/H),

where y_i_ denotes the mean value for i-th object: y_i_ = Σ_j_y_ij_/N_i_, while y denotes the overall mean: y = Σ_i_y_i_/N. In the above, M = Σ_i_N_i_ equals the total number of measurements and H = N/Σ_i_(1/N_i_) is the harmonic mean of N_i_'s.
